# Supplementary material for: Numerical techniques for design calculations of longitudinal bending in buried steel pipes subjected to lateral Earth movements
Source: R Soc Open Sci. 2019 Jul 10;6(7):181550. doi: 10.1098/rsos.181550 (PMC6689586; doi:10.1098/rsos.181550)
Supplement: Stress-Dependent Soil Modulus Subroutine [file rsos181550supp1.docx]

# Appendix A: Stress-Dependent Soil Modulus Subroutine

**SUBROUTINE** USDFLD(FIELD,STATEV,PNEWDT,DIRECT,T,CELENT,TIME,DTIME,

1 CMNAME,ORNAME,NFIELD,NSTATV,NOEL,NPT,LAYER,KSPT,KSTEP,KINC,

2 NDI,nshr,coord,jmac,jmtyp,matlayo,laccflg)

C

**INCLUDE** 'ABA_PARAM.INC'

C

C MATERIAL AND STRENGTH PARAMETERS

C

**CHARACTER***80 CMNAME,ORNAME

**CHARACTER***8 FLGRAY(15)

**DIMENSION** FIELD(NFIELD),STATEV(NSTATV),DIRECT(3,3),T(3,3),TIME(2),

* coord(*),jmac(*),jmtyp(*)

**DIMENSION** ARRAY(15),JARRAY(15)

C

C

**CALL** GETVRM('SP',ARRAY,JARRAY,FLGRAY,jrcd,

$ jmac, jmtyp, matlayo, laccflg)

**if** ((COORD(3).LE.0.05d0).and.(COORD(3).GT.-0.001d0)) **Then**

Field(1) = 1

**else if** ((COORD(3).LE.-0.001d0).and.(COORD(3).GT.-0.002d0)) **Then**

Field(1) = 2

**else if** ((COORD(3).LE.-0.002d0).and.(COORD(3).GT.-0.003d0)) **Then**

Field(1) = 3

**else if** ((COORD(3).LE.-0.003d0).and.(COORD(3).GT.-0.004d0)) **Then**

Field(1) = 4

**else if** ((COORD(3).LE.-0.004d0).and.(COORD(3).GT.-0.005d0)) **Then**

Field(1) = 5

**else if** ((COORD(3).LE.-0.005d0).and.(COORD(3).GT.-0.006d0)) **Then**

Field(1) = 6

.

.

**else if** ((COORD(3).LE.-**i**d0).and.(COORD(3).GT.-**i+1**d0)) **Then**

Field(1) = **(i+1) * 1000**

C where i = depth of soil in metres

.

.

**else if** (COORD(3).LT.-0. **Total depth of soil block (h)** d0) **Then**

Field(1) = **Last field row number that corresponds to depth (h)**

**end if**

**RETURN**

**END**
